# Cellular and Molecular Biomarkers Predictive of Response to Immunotherapy in Acute Myeloid Leukemia

**DOI:** 10.3389/fonc.2022.826768

**Published:** 2022-05-19

**Authors:** Kyle Wiatrowski, Tae Hee Kim, Amanda Przespolewski

**Affiliations:** Leukemia Division, Department of Medicine, Roswell Park Comprehensive Cancer Center, Buffalo, NY, United States

**Keywords:** acute myeloid leukaemia, immunotherapy, biomarkers, checkpoint inhibition, T cell directed therapy

## Abstract

Immunotherapy has without question revolutionized the treatment of both hematologic and solid malignancies. Over the last several years novel strategies are being developed to incorporate these groundbreaking therapies into the care of patients with AML. Here we present an overview of the recent developments in immunotherapy for AML with a focus on biomarkers of response. Topics reviewed include antibody drug conjugates, BiTEs, DARTs, checkpoint inhibitors, and cellular therapy as well as the development of biomarkers predictive of response in each class.

## Introduction

Acute myeloid leukemia (AML) is an aggressive hematopoietic stem cell malignancy anticipated to affect 20,240 Americans in 2021, with only 29.5% of patients expected to survive beyond five years (1). The standard of care induction chemotherapy for younger patients comprised of cytarabine and an anthracycline (termed 7 + 3) continues to result in high rates of mortality and relapse (2, 3). More novel therapies such as BCL2 inhibitors in combination with hypomethylating agents (HMA) appear promising, but do not yet appear to confer long-term survival benefit (4–6). Immunotherapeutic strategies offer the potential benefits of improved toxicity and durable responses (7) and are therefore being developed in AML. However, due to the heterogenous nature of AML the identification of predictive biomarkers of response are key to the further development of immunotherapy in this disease. In this review we will assess the development of various classes of immunotherapy in AML including monoclonal antibodies, T cell directed therapy and checkpoint therapy, with a focus on the emerging biomarkers that are predictive of response within each drug category (**Table 1**).

**Table 1:** Summary of Immunotherapy Biomarkers in Acute Myeloid Leukemia.

Study	Therapy	Type	Biomarker	Outcome	Commercial testing available
Castaigne et al. (8) Lambert et al. (9)	Gemtuzumab ozogamicin (GO)	Monoclonal	CD33	Prolonged EFS and RFS in high CD33+ with GO	Yes
Khan et al. (10)				Greater efficacy on relapse but not OS in high CD33+ with GO	
Fournier et al. (11)				Benefit of GO in several activating signaling mutations in AML blasts	
Ravandi et al. (12)	AMG 330	BITE	CD33	3 CR, 4 CRi, 1 morphologic leukemia free state	Yes
Godwin et al. (13)	Flotetuzumab	DART	CD123	3 CR, 2 received HSCT in primary refractory AML. Higher CD3 and CD8 cell infiltrates in baseline BM in responders	Yes
Uy et al. (14)				Median OS 10.2 months. 10-gene signature predictive of CR	
Vadakekolathu et al. (15, 16)				Increased expression of IFN-γ-related genes and immune infiltrative tumor microenvironment associated with responders	
Wei et al. (17)	Flotetuzumab + MGA012			Ongoing study	
Ravandi et al. (18, 19)	Vibecotamab	DART	CD123	2 Cr, 3 Cri, 3 morphologic leukemia free survival with overall response rate of 14%. Lower PD-1 expression on CD4+ and CD8+ T cells in responders	Yes
Assi et al. (20)	Nivolumab + idarubicin + cytarabine	ICI	T-Cell repertoire	34 (77%) CR/CRi (28 CR, 6 CRi) and 18/34 (53%) undetectable MRD. Responders had greater total infiltrative CD3+ vs nonresponders who had increased baseline CD4+ Teffs co-expressing PD-1 and TIM3	No
Daver et al. (21)	Nivolumab + azacitidine			Overall response rate was 33%, with 58% ORR in HMA-naïve patients. Increased pretherapy BM CD3+, CD4+ T effecter, and CD8+ cells; and increased peripheral CD3+ in responders	
Daver et al. (22)	Nivolumab + azacitidine+ Ipilimumab			Median OS of 7.6 months. Increasing peripheral CD8+ T cells to sites of disease over therapy duration	
Zeidner et al. (23)	Pembrolizumab + HiDAC	ICI	T-Cell repertoire	Overall response rate 46%, CR/CRi 38%. Median OS was 11.1 months. Increased frequency of senescent T cells in BM and peripheral blood and increased terminally differentiated effector T cells in peripheral blood in nonresponders. Increased TCF1+ CD8+ T cells in CR	No

## Antibody Drug Conjugate

### Gemtuzumab Ozogamicin

Gemtuzumab ozogamicin (GO) is a monoclonal antibody-drug conjugate consisting of a humanized IgG4 anti-CD33 antibody conjugated to calicheamicin (24). CD33 is primarily expressed on cells of myeloid origin, and thus on AML blasts (25). This compound has demonstrated improved outcomes in AML patients with favorable and intermediate risk cytogenetics when combined with induction chemotherapy (8, 9, 26, 27).

In the ALFA-0701 trial, 200 patients were retrospectively analyzed and split into low versus high CD33+ patients with 70% CD33+ blasts as a cutoff (8). This study did not demonstrate a difference in CR/CRp (complete remission/complete remission with incomplete platelet recovery) between low and high CD33+ patients but did show prolonged EFS (event-free survival) and RFS (relapse-free survival) in high-CD33+ patients with GO. This benefit remained with high-CD33+ patients even after adjusting for cytogenetics and *NPM1/FLT3-ITD* mutations (10). In addition, Khan et al. stratified patients by CD33+ expression, and established greater efficacy of GO in patients with higher expression of CD33+ on relapse but not on overall survival (11). Moreover, Fournier and colleagues identified that the benefit of GO is also observed in patients with activating signaling mutations within AML blasts such as *FLT3, NRAS, KRAS, JAK2*, and *PTPN11* (28). However, additional studies are needed to determine the biological role of these mutations with respect to response to GO.

## T-Cell Directed Therapy

### Bi-Specific T-Cell Engagers (BiTE)

Bi-specific T-cell engager (BiTE) therapy is comprised of a recombinant antibody constructed by two single chain variable fragments (scFvs), one binding CD3 on T cells and the other binding an antigen on tumor cells, connected by a short peptide linker (29). Agents in this therapeutic class targeting CD33 x CD3 are currently in development for patients with relapsed or refractory AML. AMG 330 is a human CD33/CD3 BiTE antibody (30). In the recent update of the Phase 1 study by Ravandi and colleagues at the 2020 American Society of Clinical Oncology annual meeting, AMG 330 appeared to be safe and tolerable in patients with relapsed or refractory AML. Of 42 evaluable patients, eight responded with 3 CR, 4 CR with incomplete hematologic recovery (CRi) and 1 patient with morphologic leukemia free state. Preliminary results showed that responders had lower tumor burden at baseline, higher CD8+ lymphocyte count, and higher Effector : Target ratio, however there was no correlation between CD33 expression on AML blasts at baseline and response (12, 31).

### Dual Affinity Retargeting Antibodies (DARTs)

Dual affinity retargeting antibodies (DARTs) are constructed using covalently linked bispecific diabody structures (32). Flotetuzumab is a humanized DART that recognizes both CD123 and CD3. The low-affinity interleukin-3 receptor α subunit (IL3RA) CD123 is expressed on the surface of up to 80% of patients with AML (33, 34). Flotetuzumab demonstrated antileukemic activity especially in patients with primary chemotherapy refractory disease versus relapse disease (13). In 6 primary refractory AML patients treated with phase 2 dose of flotetuzumab, Godwin et al. showed that responders had higher CD3 and CD8 cell infiltrates at baseline compared to non-responders. In two of the three CR patients who underwent allogenic hematopoietic stem cell transplant, flotetuzumab induced clustering around CD123 AML cells in the bone marrow (17). Non-responders were also found to have a higher expression of PD-L1 compared to those with complete response (23% vs 16%). Other studies combining flotetuzumab with MGA012, an anti-PD-1 antibody are ongoing (15).

Vadekekolathu and colleagues studied gene expression in bone marrow biopsies as well as clinical outcomes of AML patients treated with flotetuzumab to evaluate for immune-based biomarkers for response to therapy (16). In this study, 92% of patients with evidence of anti-leukemic activity from flotetuzumab were found to have an immune-infiltrated tumor microenvironment relative to non-responders prior to initiating therapy using the PanCancer IO360 gene expression assay. In addition, responding patients had a higher tumor infiltration score (TIS) than non-responders, and was a strong predictor of response. These authors also demonstrated that these biomarkers predict response in patients with *TP53* mutated AML (14). Specifically, patients that achieved CR were found to have significantly higher tumor inflammation signature, *FOXP3*, *CD8*¸inflammatory chemokine and *PD-1* expression scores at baseline as compared to non-responders. This group then went on to identify a parsimonious expression signature encompassing the top ten genes to be predictive of CR to flotetuzumab which included *FCGR3A/B, FPR1, FBP1, NOTCH2, SERPINH1, CD8B, ICOS, PDGFA, CRABP2, THBS1* (18). An analysis of functional protein association networks demonstrated that these ten genes to be enriched in ontologies and pathways associated with antigen processing and binding, vascular endothelial growth factor-activated receptor activity, NOTCH signaling, micro-RNA regulation in cancer and T helper type 1 (Th1) and Th2 differentiation. However, in this study CD123 expression on AML blasts did not correlate with response. Of the 30 patients treated on this study who experienced early relapse or who failed primary induction therapy for AML, 26.7% achieved a CR or CR with partial hematological recovery (CRh). The median overall survival of patients having achieved a CR/CRh was 10.2 months (range, 1.87-27.27), with 6- and 12- month survival rates of 75% (95% confidence interval [CI], 0.450-1.05) and 50% (95% CI, 0.154-0.846).

Another CD123 x CD3 DART, Vibecotamab is also in early development (19, 35). Initial results of the Phase 1 study of this agent in patients with relapsed and refractory (R/R) AML did demonstrate antileukemic activity. Of 51 evaluable patients, 2 achieved CR, 3 achieved CRi and two patients obtained morphologic leukemia free survival leading to an overall response rate of 14%. Responders to Vibecotamab demonstrated a lower burden of disease as well as low PD-1 expression on CD4+ and CD8+ T cells compared to non-responders. However, as with flotetuzumab, response did not correlate with expression of CD123 on AML blasts.

## Checkpoint Inhibitor Therapy

To date, many initial single-drug immune checkpoint inhibitors (ICI) in the treatment of myeloid malignancies have lacked robust clinical efficacy. CPIs in the treatment of hematologic malignancies are still being investigated, after promising applications in solid tumor pathologies such as melanoma, lung and renal cancers. Two well described negative checkpoint pathways are CTLA-4 (CD152) and PD-1 (CD279). CTLA-4 has been shown to play a role in downregulating initial T cell activation and programmed cell death-1 while PD-1 is an inhibitory receptor which when ligands (PDL-1, PDL-2) bind diminishes activated T cell and B cell responses (36). Inhibition of PD-1 and PD-L1/PDL-2 has been shown to significantly enhance anti-tumor response by generating widespread immune activation (20).

In 2019, a phase 2 open-label study conducted by Ravandi et al. examined the response of newly diagnosed AML or high-risk myelodysplastic syndrome (MDS) patients (ages 18-65) undergoing idarubicin (I) and cytarabine (A) induction with the addition nivolumab (Nivo) (21). Treatment consisted of 1-2 cycles of induction (A) 1.5 g/m^2^ (days 1-4) and (I) 12 mg/m^2^ (days 1-3) followed by (Nivo) 3 mg/kg on day 24± 2. The study concluded that the addition of Nivolumab to standard of care (I+A) has potential as a future therapeutic option with a tolerable side effect profile. Of the patients treated, 34 (77%) achieved CR/CRi (28 CR, 6 CRi) and 18/34 (53%) had undetectable MRD by flow cytometry (FC) following induction. Flow cytometry was further used to characterize T cell subset expression from BM aspirates: CD4 T effectors [Teff]: CD3+CD4+CD127lo/+Foxp3-, CD4 T regulatory:CD3+CD4+CD127-Foxp3+, and CD8 T cells. Patients who achieved CR/CRi were found to have a greater total infiltrative CD3+ T-cell population versus NR who had a significantly higher percentage of CD4 Teff co-expressing PD1/TIM3 (p<0.05), comparable to the TIM3/LAG3 phenotype that in AML that is linked to exhausted immune presentation.

Daver and colleagues published the results of a single-arm trial investigating azacitidine (AZA) and nivolumab combination therapy in relapsed/refractory (R/R) acute myeloid leukemia patients (21). The study yielded promising results as overall response rate (ORR) to therapy was 33%, with 58% ORR in HMA-naïve patients. Bone marrow aspirates identified that responders had an increased frequency of pretherapy CD3+ cells, including CD4+ T effector cells and CD8+ cells as compared with non-responders (32.5% vs. 17.5%; *P*=0.04). In addition, a higher frequency of CD3+ cells were also observed within the peripheral blood in responders as compared with non-responders. This difference within the bone marrow and peripheral blood persisted following 2 cycles of therapy. Following further analysis with 36-parameter CyTOF responders were found to have significantly less populations of Th17-like T cells as compared to non-responders (1.5% vs. 4%; *P=*0.02) within the bone marrow prior to initiation on therapy. Moreover, responders were also found to have an increased frequency of an effector CD8+ T-cell cluster expressing CD45RA^+^PD1^lo^Tbet^hi^Eomes^lo^ within pretherapy bone marrow aspirate of responders as compared to non-responders (11.2% vs. 2.5%; *P=*0.002).

The addition of Ipilimumab was hypothesized by Daver and colleagues to further alter the tumor microenvironment, bolstering PD-1 pathway escape and enhancing CTLA-4 activation. AZA+Nivo (n=59) was compared to AZA+Nivo+Ipi (n=36) in R/R AML patients, defined by ECOG ≤2 (22). Compared to HMA-control and AZA+Nivo which had a median OS of 5.9 and 4.6 months, AZA+Nivo+Ipi demonstrated a median OS of 7.6 months (P=0.01). Biomarker response was striking with the addition of Ipilimumab in responders, where the percentage of mobilized peripheral CD8+ T-cells to sites of disease (BM and EMD) was shown to longitudinally increase over the duration of therapy exposure (37).

Pembrolizumab has also been evaluated in combination with azacitidine in patients with relapsed/refractory AML as well as older (≥ 65 years old) patients with AML (23). Within the relapsed/refractory cohort, 37 patients were treated and 78% (n=29) were evaluable for response. Four patients (14%) achieved CR/CRi, and one patient achieved PR (4%). Four patients experienced hematologic improvement (HI) (14%) and seven patients had stable disease (SD) (26%). The median OS for the whole cohort, responders + SD, and CR/CRi/PR was 10.8 months (40% 1-yr), 13.9 months (51% 1-yr), and 17.2 months (75% 1-yr). The median event-free survival (EFS) was 6 months, with a median disease-free survival (DFS) for CR/CRi patients is 8.5 months. Within the newly diagnosed cohort, twenty-two patients were treated and seventeen were evaluable for responses. A total of eight patients achieved CR/CRi (6/2) (47%), two patients experienced PR (12%), two patients had HI (12%), and 4 patients were found to have SD for at least 6 cycles (24%). The median OS for the whole cohort was 13.1 months, for responders + SD 13.4 months (70% 1-yr) and not reached for patients with CR/CRi/PR (79% 1-yr) with a median follow up of nine months. The median EFS was 9.6 months, with median DFS for CR/CRi patients being 16.6 months. The authors did describe cytogenetic and molecular profiles associated with response to therapy, however there were no significant biomarkers identified.

Recently, Zeidner et al. evaluated the combination of high dose cytarabine (HiDAC) chemotherapy followed by the anti-PD-1 monoclonal antibody pembrolizumab in patients with relapsed or refractory AML (38). Within the study, the overall response rates (ORR) and rates of CR/Complete response with incomplete platelet recovery (CRi) were 46% and 38% respectively. Median OS was 11.1 months (95% CI, 6.3-13.9 months). Median event-free survival (EFS) and relapse-free survival were 6.7 months (95% CI, 4.9-11.1 months) and 5.8 months (95% CI, 2.2-10.4 months). Furthermore, progression-free survival was 5.7 months (95% CI, 1.9-10.4 months). Correlative studies were performed on treated patients which identified that patients who were non-responders were found to have a significant increase in the frequency of senescent T cells (CD45RA+, KLRG1+, CD57+) within the peripheral blood and bone marrow as compared to responders. In addition, non-responders had a significant increase of terminally differentiated effector T cells in the peripheral blood as compared to responders. Furthermore, a significant increase in populations of CD8+CD45RA-CD27+/intCD28+PD1+TCF+ T cells prior to therapy was identified in patients who achieved CR as compared with non-responders.

Saxena and colleagues evaluated avelumab in combination with azacitidne in patients with relapsed and refractory AML (39). A total of nineteen patients were treated on this study with an overall complete remission rate of 10.5%. Both patients with CR were found to have residual thrombocytopenia. The median overall survival of treated patients was 4.8 months. Bone marrow blasts were analyzed by mass cytometry and were found to have increasing PD-L2 expression on therapy. However, given the limited clinical activity of this regimen, the significance of this finding is unclear.

Most recently, Amer Zeidan et al. evaluated azacitidne with or without durvalumab in elderly patients with newly diagnosed AML (40). Patients were randomized to first-line therapy with azacitidine (Arm A, n= 64) or without (Arm B, n=65) durvalumab. Overall response rate (CR] + CRi) was similar in both arms (Arm A, 31.3%; Arm B, 35.4%), as were overall survival (A, 13.0 months; B, 14.4 months) and duration of response (A, 24.6 weeks; B, 51.7 weeks; P=0.0765). No new safety signals emerged with combination treatment. Correlative studies including DNA methylation, mutational status, and PD-L1 expression were performed, however none were found to be associated with response to treatment.

As we continue to elucidate the biological mechanisms of AML progression and relapse, one proposed disease escape mechanism is through phagocytosis inhibition of innate immune system macrophages. CD47, a surface immunoglobulin that identifies self and has been coined as a “do not eat me signal,” has been identified as being overexpressed in leukemic cell lines (41).

Magrolimab, an anti-CD47 macrophage checkpoint inhibitor, is currently being investigated for combination use in leukemia patients. Sallman et al. reported the results of the first-in-class phase 1b trial examining magrolimab in combination with AZA in AML patients. The foundational study showed that magrolimab + AZA has a similar safety profile as AZA alone and overall was well-tolerated. Initial findings helped spur interest in other potential combination regimens; when of the patients evaluable for efficacy (n=34), 65% achieved an OR with a time to response of 2.04 months. Strikingly, *TP53* mutant patients (n=21 of those evaluated for efficacy) had an OR (71%) and a CR/CRi (48%/19%). Overall survival amongst total *TP53* mutant patients (n=34) and *TP53* wild type patients (n=16) was 12.9 and 18.9 months respectively (42).

At the 2021 American Society of Hematology Annual Meeting, Daver et al. presented results from a phase 1b/2 trial to study the side effects and best dose of the triplet combination AZA, venetoclax (VEN), and magrolimab in high-risk AML patients. The dose schedule was AZA (75 mg/m^2^ D1-7), VEN (400 mg D1-28), and magrolimab during the first 28-day 1 D1/4/8/11/15/22; weekly in cycle 2, and every 2 weeks in cycles after that. Secondary objectives of the study included CR/CRi rate and OS. The three treatment arms were stratified between newly diagnosed (ND) (n=23), R/R VEN-naïve AML (n=8), and R/R post-VEN failure AML (n=13). The most promising efficacy of the triplet therapy was in newly diagnosed high-risk patients, notably ND TP53–wt (ORR: 63%, mOS 18.9m) and *TP53* mutant AML (ORR 69%, mOS 12.9m). Among all ND patients, CR/CRi was high (94%) (43). At the time of publication, the study authors did not make any inferences or suggestions as to correlative biomarker responses to therapy amongst the studied groups.

Vyas et al. described an ongoing phase 2 open-label study (NCT04778410) that include three phase 1 cohorts: cohort 1 (first-line ≥75 years or 18-74 years not suitable for intensive chemotherapy (IC)) to receive a combination of magrolimab/VEN/AZA, cohort 2 (R/R AML after initial IC) receiving magrolimab with mitoxantrone, etoposide, and cytarabine (MEC), and cohort 3 (as maintenance ≥55 years old having achieved a CR/CRi with MRD positivity *via* FC, after IC, and not be suitable for HSCT) being administered magrolimab with oral AZA (CC-486). The primary efficacy endpoints in cohort 1 and 2 is the CR/CRi rate, with secondary endpoints examining OS, ORR, and MRD-negative CR/CRi rates. The primary efficacy endpoint Cohort 3 is the MRD-negative CR/CRi rate. Each cohort is planned to enroll 6 patients, whereby magro will be administered on the same cycle across cohorts, along with the anticipated combination interventions (44).

Lastly, Daver et al. detailed a phase 3, randomized, open-label interventional trial specifically examining TP53-mutant AML patients. This randomized trial follows the previously described Sallman et al. first-in-class phase 1b study. The study is still accruing patients but is targeting approximately 346 patients that will be randomized (1:1) to either magrolimab+AZA or given physician evaluation of patient fitness to VEN+AZA or 7 + 3 chemotherapy. Recruited patients will be treatment naive ≥18 years old with histologically confirmed AML and at least one TP53 gene mutation or biallelic 17p deletions. Intervention administration will consist of magrolimab priming dose (1 mg/kg D1 and 4, 15 mg/kg D8, and 30 mg/kg D11,15,22) during the first 28-day cycle. In cycle 2, magrolimab (30 mg/kg) will be administered weekly, followed by every other week cycle 3+. The crucial primary endpoint is OS in patients receiving non-intensive therapy, and the key secondary endpoint is OS in all patients (45). As CD47-based checkpoint inhibitors are early in development, there remains a gap in the literature investigating the clinical impact of magrolimab and biomarker responses. Future publications consisting of phase 3 clinical data will help to unveil future magrolimab combination therapy uses.

## Cellular Therapy

Given the durable responses of cellular based therapy in patients with acute lymphoblastic leukemia (46, 47), there has been great enthusiasm for the development of cellular therapy for patients with AML. Autologous CAR-T therapy targeting a variety of antigens including CD33 are in development. However, results to date have been disappointing. Tambaro and colleagues have described their experience with the Phase I clinical trial evaluating the safety and efficacy of a CD33 targeted CAR with 4-1BB and CD3ζ endo-domains that have been co-expressed with truncated human epidermal growth factor receptor (HER1t) in patients with relapsed and refractory AML (48). Unfortunately, of the eight patients that were enrolled and were able to undergo apheresis, only three were able to receive product as product for four of the patients failed to meet pre-specified criteria for release and two patients had rapidly progressive AML. None of the patients who did received infusion achieved a response.

Another antigen recently engineered includes the Natural Killer Group 2D (NKG2D) receptor, which targets NKG2D ligands that are expressed on AML blasts (49–52). CYAD-01 is a first generation NKG2D CAR-T which was evaluated in patients with relapsed or refractory high risk MDS and AML (53). Several patients were treated in this Phase I study, however only three patients experienced clinical benefit. Further modifications to this product include changes to improve persistence and expansion in patients, with patient enrolment ongoing (53).

CD123 specific CAR-T cells are also under investigation to treat AML. Wermke and colleagues are evaluating a second-generation CAR-T platform using a CD28 costimulatory domain (54). This first in human product is being investigated in patients with relapsed or refractory AML that have ≥ 20% CD123 positivity on AML blasts. A total of three patients to date have been treated. Notably, the maximum amount of disease burden within these patients was 30% blasts. One patient achieved a partial response, and the remaining two patients achieved CRi. Despite the small number of patients treated, response appeared to occur irrespective of the degree of CD123 expression with one patient who achieved CRi only having 26% of blasts expressing CD123.

Challenges related to the employment of this class of therapy in patients with AML include the aggressive nature of relapsed and refractory AML which requires the need for expeditious product generation, as well as inability to acquire sufficiently product due to frequent lymphopenia in this patient population (48, 55). This has led the field to shift the development of cellular therapy in this patient population to include strategies to mitigate those challenges including “off the shelf” (NCT04310592, NCT03190278) and donor-derived engineered products for those patients undergoing allogenic hematopoietic stem cell transplant (NCT04679194).

Although this is a field met with great enthusiasm, there are no clear biomarkers of response to cellular therapy that have emerged to date in AML patients. This is likely due to the small number of patients treated to date and slower cadence of development compared to other disease. However, an emerging trend among responding patients appears to be a lower burden of disease as compared to non-responders. In addition, there is a requirement on most protocols for eligible patients to express the antigen of interest, but a clear threshold of expression resulting in response is unknown.

## Conclusions and Future Directions

Over the last ten years, immunotherapy has revolutionized the treatment and outcomes of cancer patients. The role of immunotherapy in the treatment of patients with AML has come to light more clearly in the more recent few years. These studies evaluating various immunotherapeutic approaches remain in the very early stages with limited numbers of patients, although the results of these studies are encouraging with signals of efficacy.

Biomarkers play a critical function in identifying appropriate patients for immunotherapy- based clinical trials in AML. These include circulating and bone marrow-residing CD4+ and CD8+ T cell subsets, quantity of target antigen expression, as well as gene expression profiles of immune subsets. These subsets of T-cells and myeloid cells can be identified through common modalities such as flow cytometry, however, the sample acquisition and analysis should be carried out in a central laboratory setting. There are nuances required in identifying the various subsets and inter-operator variability. In contrast, biomarkers such as the common cell surface markers CD33 and CD123 could be carried out at local laboratories since these markers are widely used.

From our current understanding, AML patients require a functional T cell compartment to benefit from immunotherapy. Given this, it is possible that patients may have a higher likelihood to obtain benefit from immunotherapy earlier on in the disease course as opposed to following several lines of therapy for released disease. Furthermore, as the literature reviewed here has demonstrated, biomarkers can identify patients within traditionally poor risk categories such as *TP53 mutant* AML that can respond to immunotherapy (56). This underscore the importance of further developing and implementing biomarkers in the treatment algorithm for these patients as responses can be achieved in this traditionally difficult to treat patient population.

Molecular changes within AML represent an area in need of further development as biomarkers for immunotherapy in this disease. Interestingly, certain mutations within AML can be immunogenic, and are being investigated as therapeutic targets using cellular therapy. For example, *NPM1* mutations have been found to elicit endogenous T-cell immunity and *NPM1* specific T-cell responses (57–60). On the contrary several mutations have been associated with dysfunctional immune responses in AML. For example, *JAK2 V617F* mutation has been shown to result in leukemia cell intrinsic upregulation of PD-L1 (61). In addition, *RUNX1* mutation can modulate NF-kb signalling *via* a cell intrinsic manner and has promoted inflammatory signalling within the bone marrow microenvironment (62). In gliomas, the *IDH1* mutation has a negative impact on the immune system resulting in reduced Natural Killer ligands and decreased tumor lysis, as well as decreased chemokines CXCL9, CXCL10 with subsequent reduction in the number of CD3+ CD8+ tumor infiltrating lymphocytes (63, 64). Although *IDH1* is present in some patients with AML, the impact on the immune microenvironment has yet to be fully elucidated.

Moreover, it is crucial that detailed correlative science takes place in the context of these clinical trials developing immunotherapy for AML. A thorough assessment of the immune repertoire is required and can be achieved through the use of techniques such as single-cell RNA sequencing, mass cytometry and single cell cytokine analysis. Obtaining biomarkers with this level of detail will enable proper patient selection, as well as identify combinatorial approaches for immunotherapy in AML.

## Author Contributions

KW, THK, and AP performed literature search and drafted the article. Critical review of the article was performed by AP. All authors contributed to the article and approved the submitted version.

## Conflict of Interest

The authors declare that the research was conducted in the absence of any commercial or financial relationships that could be construed as a potential conflict of interest.

## Publisher’s Note

All claims expressed in this article are solely those of the authors and do not necessarily represent those of their affiliated organizations, or those of the publisher, the editors and the reviewers. Any product that may be evaluated in this article, or claim that may be made by its manufacturer, is not guaranteed or endorsed by the publisher.
